# Changes in Employment Uncertainty and the Fertility Intention–Realization Link: An Analysis Based on the Swiss Household Panel

**DOI:** 10.1007/s10680-016-9408-y

**Published:** 2017-02-24

**Authors:** Doris Hanappi, Valérie-Anne Ryser, Laura Bernardi, Jean-Marie Le Goff

**Affiliations:** 1 LIVES—Swiss National Centre of Competence in Research, University of Lausanne, Bâtiment Géopolis, 1015 Lausanne, Switzerland; 2 Department of Demography, University of California Berkeley, 2232 Piedmont Ave, Berkeley, CA 94720-2120 USA; 3 FORS, Swiss Center of Expertise in the Social Sciences, c/o University of Lausanne, Bâtiment Géopolis, 1015 Lausanne, Switzerland; 4 LINES—Life Course and Inequality Research Center, University of Lausanne, Bâtiment Géopolis, 1015 Lausanne, Switzerland; 5 Institute of Social Sciences, University of Lausanne, Bâtiment Géopolis, 1015 Lausanne, Switzerland

**Keywords:** Fertility intentions, Employment uncertainty, Life course, Gender, Panel data, Switzerland

## Abstract

How do changes in employment uncertainty matter for fertility? Empirical studies on the impact of employment uncertainty on reproductive decision-making offer a variety of conclusions, ranging from gender and socio-economic differences in the effect of employment uncertainty on fertility intentions and behaviour, to the effect of employment on changes in fertility intentions. This article analyses the association between a change in subjective employment uncertainty and fertility intentions and behaviour by distinguishing male and female partners’ employment uncertainty, and examines the variation in these associations by education. Using a sample of men and women living in a couple from the Swiss Household Panel (SHP 2002–2011), we examine through multinomial analysis how changes in employment uncertainty and selected socio-demographic factors are related to individual childbearing decisions. Our results show strong gendered effects of changes in employment uncertainty on the revision of reproductive decisions among the highly educated population.

## Introduction

Recent below-replacement fertility has prompted an animated debate among demographers seeking a better understanding of childbearing intentions. Recent studies have found intentions to be a powerful predictor of fertility at the aggregate level (Morgan and Rackin 2010; Quesnel-Vallee and Morgan 2003; Liefbroer et al. 2015). At the individual level, however, intentions do not always match actual outcomes, as the authors above indicate. There is a consensus among scholars that low total fertility (with the total fertility rate—TFR—below 1.5) results from obstacles that intervene between the intention to have children and their realization. Employment uncertainty is one such obstacle. The fear of losing one’s job and becoming unemployed while establishing one’s career are among the main obstacles to realizing childbearing intentions.

Previous studies, however, offer a variety of arguments for the intention–realization gap. First, the effect of employment uncertainty on fertility may differ between partners; yet, most studies focus on women’s childbearing decisions made despite the employment uncertainty of their partners (for exceptions, see Kravdal 2002; Gebel and Giesecke 2009). Second, objective employment uncertainty (e.g. unemployment, precarious contracts) is in the same way associated with fertility as perceived employment uncertainty (e.g. fear of losing one’s job), which is in itself a deterrent to childbearing (Golsch 2003; Bernardi et al. 2008; Kreyenfeld and Konietzka 2014; Hofmann and Hohmeyer 2013). Third, the association between employment uncertainty and fertility may differ depending on socio-economic resources. Often, employment uncertainty generates high opportunity costs in terms of forgone career promotions or salary increases among highly educated populations and therefore results in delayed parenthood and fewer births in this group (Rindfuss et al. 1996; Martin 2000; Adsera 2004; Blossfeld et al. 2005; Kravdal and Rindfuss 2008; Pailhé and Solaz 2012). Fourth, both employment uncertainty and fertility intentions may be differentially unstable over time depending on employment status. The latter has been examined by Spéder and Kapitány (2009), who show that unemployed men in Hungary were more likely to abandon childbearing intentions than those who were employed.

This article identifies the way in which changes in employment uncertainty are linked to changes in fertility intentions and behaviour over a 2-year period, by linking uncertainty to intention trajectories (e.g. keep intending to have a child, abandoning or postponing an intention to have a child). The analysis distinguishes between male and female partners’ changes in perceived employment uncertainty, and examines differences in these associations by education levels. The analysis also includes fertility intentions of both members of a couple, since research has confirmed that partners’ disagreement leads to substantial delay in childbearing (Testa et al. 2011). Our analysis addresses two central research questions. First, what are the effects of a rise and decline in male and female employment uncertainty on fertility intentions and their realization? Second, to what extent do effects of employment uncertainty vary according to education level?

To address our questions, we use panel data from ten waves of the Swiss Household Panel (SHP 2002–2011). The SHP contains information about short-term fertility intentions in each wave starting from 2002. Drawing on Spéder and Kapitány’s analyses (2009), we explicitly focus on short-term fertility intentions, which refer to having a child within two years and the follow-up on an intention during the respective time period, i.e. intended births. Our analyses focus on partnered men and women of reproductive age, thus covering the relationship between their fertility intentions and each partner’s employment uncertainty. Employment uncertainty as analysed here refers to an employed person’s *assessment of how secure his or her job is,* or *how likely he or she is to lose the job in the near future*, which is in line with the definition of ‘cognitive’ job insecurity (Anderson and Pontusson 2007; Esser and Olsen 2011).

The study context is Switzerland, a country where remarkably low-fertility rates (e.g. the TFR was 1.54 according to OFS data of 2014) and high rates of childlessness correlate with highly gendered labour market participation. While men work almost universally in full-time jobs, women, whose labour force participation is high, work mostly part time (Sobotka and Zeman 2011; Levy et al. 2006). Part-time jobs are often more precarious, often associated with low income (Charles 2011) and restricted options to contribute to retirement pensions, which mostly concerns women with children (Liebig et al. 2015). In addition, in Switzerland work–family reconciliation policies are poor: childcare is extremely expensive and its provision insufficient to meet the demand, and paternity and parental leave do not exist at the federal level (Valarino and Bernardi 2010). In such a context, perceived employment uncertainty may be due to worries of losing a job even if on a permanent contract, or due to (especially women’s) worries of not being able to balance work with childrearing and care duties.

## Theoretical Background

Since the 1980s, job insecurity has become an inherent characteristic of adult life, and this has long-term implications. Main biographical events in this life stage include entry into and establishment in the labour market, organizing and managing one’s own career, and the birth of one’s first child. Increasing employment uncertainty during the recession years and cyclical economic upturns have made these processes more complicated (Blossfeld et al. 2005). As a consequence, an individual’s time and resources, which would otherwise be invested elsewhere, are put towards efforts to maintain or re-establish one’s position in the labour market. Childrearing suffers fierce competition in such situations of scarce resources (McDonald 2000; Voydanoff 2005; Philipov 2009). The difficulty to combine the roles of parent and employee is seen as a major reason for the postponement of fertility observed in most advanced Western societies nowadays (Matysiak and Vignoli 2008; Blossfeld and Hofmeister 2007; Kreyenfeld et al. 2012).

Time incompatibility is often resolved by a sequential ordering of events, which means that some events get postponed. One example of the ordering of events is the sequence of stabilizing one’s labour market position followed by a first birth (Bernardi et al. 2008). This sequence is due, on the one hand, to the widespread assumption that stabilizing one’s labour market position is a way to ensure the material resources and work autonomy required to care responsibly for one’s child (Begall and Mills 2011). On the other hand, institutions regulating the education and labour market transitions are more rigidly organized around strict age schedules in early young adulthood in comparison with parenthood: no institution is proclaiming one must become a parent by a particular age. The consequence is that the postponement of births until the labour market position is certain is a shared social norm, especially in contexts where incompatibility of work and family is high (Hochschild and Manchung 1989).

The way individuals respond to employment uncertainty is likely to depend on the welfare system, as well as on the education level and the prevalent attitudes towards work. The extent to which employment uncertainty constrains a person’s ability to accommodate work around childcare and sustain a family is decisive in this response (Voydanoff 2005). A number of empirical studies confirm the negative impact of uncertainty on fertility by education (Kohler and Kohler 2002; Fiori et al. 2013; Kreyenfeld 2009). Even though anticipations are hard to make, employment aspirations and the opportunities to combine work and family life expand along with rising education levels (Korpi 2000). On the one hand, highly educated persons are generally better able to collate the resources they need to avoid risks resulting from employment uncertainty, and they usually return to the labour market more quickly after childbirth (Liefbroer and Corijn 1999; Adsera 2011). This group is less vulnerable to economic downturns and times of high unemployment. On the other hand, *human capital* theorists suggest that since highly educated individuals face higher opportunity costs of childbearing, they make fertility decisions more deliberately (Spéder and Kapitány 2009). They may develop work-oriented lifestyles (Berrington 2004) and be more likely to abandon their fertility plans if their employment is uncertain or if professional advancement competes with childbearing. A Swiss study showed that young low-educated women tend to choose an apprenticeship or a formation oriented towards a professional career that allows the reconciliation between family and work (Gianettoni et al. 2015). Studies show also that a lot of women, yet, at least temporarily withdraw from the labour market, before they eventually return part time to it (Levy et al. 2006). Such a traditional family model increases the likelihood that fertility intentions and behaviour in this group vary strongly in relation to the relatively unfettered exposure of the main income provider to the economic climate. Education may have a direct impact on the link between childbearing intentions and subsequent behaviour, because the ability to pursue one’s own plans and to overcome obstacles that impede intentions from being realized could depend on informal support networks that differ according to education level (Rossier and Bernardi 2009).

The response to employment uncertainty in adult lives implies the existence of a decision-making process (Blossfeld and Hofmeister 2007). Intentions are a main component of the irreversible decision whether or not to have a child (Miller and Pasta 1994; Johnson-Hanks 2005). Several studies, however, show that the fertility levels forecasted by intentions do not match actual fertility (Toulemon and Testa 2005; Quesnel-Vallee and Morgan 2003), as fertility goals are generally over- or underachieved or change over time. While some thus attributed a minor role to fertility intentions, for instance at the time of the baby boom when modern contraception was not broadly diffused and the TFR exceeded the level of intended births (Quesnel-Vallee and Morgan 2003; Bongaarts 2002), other scholars have concluded that change in fertility intentions over time helps to better understand why people revise and especially abandon their fertility plans (Léridon 1995). The better we understand changes in intentions, the better we will understand the corresponding behaviour. Intentions are considered antecedents of behaviour in Ajzen’s theory of planned behaviour (TPB) (Ajzen and Klobas 2013). This model has been applied to several behaviours including fertility behaviour (Liefbroer et al. 2015). In contrast to the broader concept of ‘intended family size’ (often called lifetime intentions) and the intention to have any more children at all, short-term intentions tell us something about the timing of childbirth (Philipov and Bernardi 2011). Respondents to fertility surveys might, over a period of 2–3 years, become clearer about their personal life conditions and any obstacles that prevent them from realizing their intentions. Short-term intentions are therefore argued to be strongly linked to external conditions and to changes in these conditions (in our case changes in employment uncertainty); that is, people may adapt an intention to have a child in the course of two to three years by postponing or abandoning childbearing. We would like to add that one shall exercise caution when using short-term intentions to estimate the incidence of realization or the effects of life conditions on non-realization; in such cases, realizations might be underestimated and the effect of life conditions overestimated (Schoen et al. 1999; Berrington 2004).

**Fig. 1:**
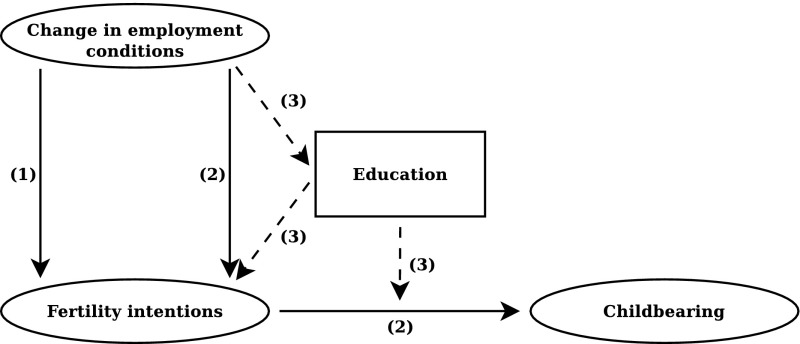
Schematic representation of the relationship between employment uncertainty, fertility intentions, and childbearing

According to Blossfeld and Hofmeister (2007), individuals who experience employment uncertainty (such as worries about losing one’s job) may delay childbearing. More precisely, employment uncertainty undermines the intention to have a child in two ways: it may have a direct effect on its formation or it may hinder its realization. These links are presented graphically in Fig. 1. Line (1) presents the impact of changes in employment uncertainty on the construction of intention trajectories (such as maintaining positive intentions over time, abandoning an intention, and postponing an intention), and Line (2) shows the link between an intention to have a child and its realization.

Consider the first case where we examine the fertility *intention trajectory* (Line 1) under perceived employment uncertainty. Suppose that an individual wants to have a child without postponement and experiences employment uncertainty. In this case, the individual will face conflicts of time and energy, and unpredictable material resources. Such conflict and the limited capacity to secure family income can be resolved by abandoning the intention to have a child, i.e. prioritizing one life domain over the other, or by postponing childbearing until later years, i.e. sequencing transitions. In this case, the individual is expected to change an intention to have a child as follows: a person may abandon an intention to have a child (the *abandoner* group) or may postpone such an intention (the *postponer* group). Alternatively, if a person experiences a reduction in employment uncertainty and has not yet reached the personal fertility goal, this person is expected to continue intending to have a child (the *stable yes* group).

In the second case, we examine the *intention*–*realization link* (Line 2) under perceived employment uncertainty. In the empirical analysis that follows, we consider employment uncertainty as a condition to the construction and realization of an intention to have a child. For the sake of simplicity in the schematic representation, changes in employment conditions referring to a rise and decline in employment uncertainty are included in the same block in Fig. 1.

Moreover, using the *human capital theory*, we examine the *education argument* (Line 3) that suggests education increases the opportunity costs of childbearing, and that it is a main deterrent in the intention to have a child, as well as its realization. Purveyors of human capital theory argue that the more educated people are, the higher the opportunity costs of realizing their fertility intentions are, and these costs are difficult to recuperate at later stages of life (Rondinelli et al. 2010). Consequently, facing individual employment uncertainty will interfere in the more deliberate childbearing decisions of the highly educated group and most likely hamper the intention to have a child, and thus, subsequent childbearing. In this group, establishing oneself on the labour market is expected to compete more strongly with responsibly caring for children even if highly educated persons often have stronger support networks (Rossier and Bernardi 2009). In the case of low-educated people, their fewer personal resources make them sensitive to general changes in economic climate and overall job insecurity, rather than to changes in the individual employment uncertainty. We thus distinguish highly educated from low- and medium-educated individuals when we analyse the link between employment uncertainty and fertility intentions and behaviour.

## Hypotheses

Based on the theoretical framework outlined above, we argue that worsening employment conditions facilitate the postponement or abandonment of the intention to have a child (*intention–trajectory argument)*, and certainly make it less probable that the intention be realized (*intention*–*realization argument*). Employment conditions that are improving make it more likely that the intention to have a child be maintained (*intention*–*trajectory argument*), or realized by the end of the two-year period (*intention*–*realization argument*). Education should be decisive in how individuals make childbearing decisions under worsening or improving employment conditions (*education argument*). According to our conceptual background, childbearing intentions always refer to a period of 2 years.

The main research task in this article is to test the hypotheses derived from these three arguments when changing employment conditions refer to the rise and decline in individual employment uncertainty. The hypotheses fit the prevailing labour market and family–work reconciliation policies and conditions in Switzerland. A vast body of literature finds that adverse employment conditions restrain childbearing plans and impede childbearing (Pailhé and Solaz 2012; Schmitt 2012; Blossfeld et al. 2005; Sobotka et al. 2011; Kreyenfeld 2009; Hofmann and Hohmeyer 2013; Özcan et al. 2010; Schneider 2015), and Switzerland is no exception (Hanappi et al. 2016; Le Goff 2005). Therefore, the above statements can be specified for the case of rising versus declining employment uncertainty, as follows:

- A rise in employment uncertainty makes it more likely that an intention to have a child within the short-term be postponed or abandoned.
- A decline in employment uncertainty makes it more likely that an intention to have a child within short term be constructed at the end of the 2-year period.
- An intention to have a child is less likely to be realized when employment uncertainty rises within the same time period.
- An intention to have a child is more likely to be realized when employment uncertainty declines within the same time period.
- Changes in employment uncertainty [(1a), (1b), (2a), and (2b)] have a strong impact on the relationship between fertility intention and its realization among highly educated individuals.
- Changes in employment uncertainty [(1a), (1b), (2a), and (2b)] have a weak impact among low- and medium-educated individuals.

The three pairs of hypotheses are expected to hold for the uncertainty effects of men’s as well as of women’s employment. However, building the hypotheses of employment uncertainty effects on fertility intentions and behaviour has to take into account differences in gender roles. When men work under rising employment uncertainty, their primary breadwinner role as well as their prospects of fathering children is threatened (Modena and Sabatini 2012; Philipov 2009; Sobotka and Testa 2008; Neyer and Rieck 2009). Men’s opportunity costs are low in Switzerland because of the prevalence of traditional gender roles in the family: men engage less in household chores than women. Hence, men’s rising employment uncertainty can be considered as threatening fertility intentions and childbearing. Most women usually work for pay and at the same time do most of the household chores; so their opportunity costs are high. Hence, women’s rising employment uncertainty threatens the intention of having a child, but at the same time makes childbearing and the related social rewards an attractive option. Declining employment uncertainty should have no gendered effects, because the (secondary) income supports the family as well; therefore, hypotheses (1b), (2b) and the corresponding (3a) and (3b) should hold true for both men and women. The remaining hypotheses for men’s and women’s intentions and behaviour differ:

- A rise in male employment uncertainty makes an intention to have a child within two years likely to be postponed or abandoned. With respect to a rise in female employment uncertainty, we expect this association to be weaker.
- An intention to have a child is less likely to be realized when male employment uncertainty rises within the same time period. An intention to have a child within the following two years is more likely to be realized when female employment uncertainty rises within the same time period.

Women’s opportunity costs can be compensated by various factors such as social support by family and friends in childrearing; child allowances or maternal leave could be attractive particularly to women with lower education (Friedman et al. 1994). In these and similar situations, rising women’s employment uncertainty may emerge as facilitating rather than constraining not only behaviour, but also intentions.

## Data and Method

### Swiss Household Panel (SHP) and Sample

We use data from the Swiss Household Panel (SHP) for 2002–2011 (Tillmann et al. 2016). The SHP is a national representative survey that combines household data with individual information on demographic events, fertility intentions, and employment-related indicators. Since 1999, this survey follows on a yearly basis all individuals within private households in Switzerland, whereby household members aged 14 years and older are interviewed. Survey attrition in the SHP is moderate concerning demographic and socio-economic variables (Voorpostel and Lipps 2011). The non-response bias is also rather low (Lipps 2006).

We included in our sample men and women living in a partnership because childbearing intentions might have been biased if a given respondent had no partner at the time of the interview (Berrington 2004; Voas 2003; Philipov et al. 2006; Neyer et al. 2013). We randomly selected one of the partners for our multivariate models (i.e. male or female partner). Since our analyses required that information be available on both partners’ perceived employment uncertainty, we selected men and women who were active on the labour market from the time of the first interview until 24 months after the interview. This resulted in a sample of 1634 individuals, among them women aged 22–45 and men aged 22–55 at the time of the interviews. Very few of the interviewed men and women outside these age ranges declared their fertility intentions. We focus on intentional childbearing, wherein the underlying principles differ from the dynamics of unintended births (Williams 1991).^1^ We are aware of possible selectivity bias from excluding this subcategory a priori; however, we found no viable way of including such couples in the study. The number of unintended births in the SHP data has on average been only six per year between 2003 and 2011, based on available information about prior intentions.^2^

### Dependent Variable

The dependent variable was constructed relying on Spéder and Kapitány’s classification (Spéder and Kapitány 2009). We use three rubrics to construct five fertility intention/behaviour trajectories, distinguishing intention stability, intention revision, and intention–realization: (1) whether the respondent has the intention to have a child within the 24 months following wave *n*, based on the question ‘Do you intend to have a child in the next 24 months?’; (2) whether the individual had a child during the 24 months between wave *n* and wave *n* + 2; and (3) whether the individual intends to have a child in wave *n* + 2 if s/he did not have a child between wave *n* and wave *n* + 2. Twenty-six per cent of the sample intended to have a child within two years, while 51% actually had a child by the time of the second wave (cf. Table 1; 93.5% of short-term intentions remained stable while the remaining 7.5% changed in wave *n* + 2). We created our dependent variable based on this information. The first group is composed of individuals who intended to and did in fact have a child within 24 months; this group is called the *intended parents* group. Respondents who intended to have a child in wave *n* but did not have a child within 24 months are differentiated according to their intention regarding wave *n* + 2: individuals who maintained a positive intention are classified as the *stable yes* group (the second group), and those who abandoned their intention are labelled *abandoners* (the third group). The next category includes respondents who did not intend to have a child at the time of the first interview on intentions: individuals who changed their intention and wanted a child in wave *n* + 2 are classified as *postponers* (the fourth group). Finally, the fifth group comprises individuals who did not intend to have a child within 24 months in either wave *n* or wave *n* + 2, and did not have a child within this time frame: this group is labelled the *stable no* group. Since we have specified five trajectory types regarding fertility decisions, several comparisons could be made. We focus our research on what determines the stability of a positive fertility intention as well as a lack thereof (the *stable no* group) and also attempt to distinguish those who maintain their intention (the *stable yes* group) from those who give up on their intention (*abandoners*) within the 24-month observation window.

**Table 1:** Observed fertility intention–realization types in the Swiss Household Panel by level of education

Intended to have a child within 2 years at wave *n*	Had a birth between waves *n* and *n* + 2	Intended to have a child at wave *n* + 2	Sample size (*N*)			Type of fertility intention–realization type
			L–M	H	*N* total	
Yes	Yes		114	94	238	Intended parents
Yes	No	Yes	79	67	146	Stable yes
Yes	No	No	34	29	63	Abandoners
No	No	Yes	40	32	72	Postponers
No	No		705	440	1145	Stable no

L–M, low–medium education; H, high education

Our respondents entered our sample when they first declared a given fertility intention (wave *n*); we followed up on them after 24 months, in the next two SHP waves. According to how we constructed our dependent variable, the window of observation was a maximum of 24 months (wave *n* until wave *n* + 2). For all respondents, we looked at whether they had a child within the 24 months following the first declared fertility intention: if they had a child within this time frame, our observation ended with the event of the childbirth; if not, their intention in wave *n* + 2 entered our analysis, allowing us to test stability and change in intention to have a child after the 24-month period. Given the correlation of intentions between partners in the same household, our measure of the male or female respondent’s intention can also be considered as the couple’s intention to have a child. Descriptive information on the measures is given in Table 1.

### Explanatory Variables

#### Changes in Partners’ Employment Uncertainty

Male and female partners were asked, ‘Would you say that your job is very secure, quite secure, a bit insecure, or very insecure?’ This information was complemented by perceived unemployment risk based on the question: ‘How do you evaluate the risk of becoming unemployed in the next 12 months?’ For the very few respondents for whom neither of the two survey answers were applicable, we added those having a limited contract of less than three years to the uncertain group (for similar approaches, see Blossfeld et al. 2005; Golsch 2003). All temporary contracts together account for no more than 5% in the SHP.^3^ Workers on time-limited contracts usually assess their jobs as less secure; they are also more worried than other employees about becoming unemployed. These worries are based on the fact that time-limited contracts are often used by employers to adjust the workforce size to comply with the demand for labour. When the latter decreases, for example in times of crises, contracts are not renewed (Kalleberg 2009). Switzerland is no exception (Greppi et al. 2010): contracts shorter than three years are mostly tied to specific productivity targets, and neither provide workers any form of stability beyond the expiration of the contract, nor grant any work–family reconciliation measures. Our cross-sectional investigations show that time-limited contracts concern mostly women, take the form of replacements jobs, like those due to maternity leaves, and are concentrated in the health and service sectors.

We dichotomize the uncertainty variable and distinguish changes in employment uncertainty from stability. We compute two dummy indicators to capture the direction of change between wave *n* and wave *n* + 2. The first dummy variable identifies partners whose employment conditions deteriorated over time, where 0 means no change and 1 means a rise in employment uncertainty. The second dummy variable identifies those whose employment conditions improved over time, where 0 means no change and 1 means a decline in employment uncertainty.

As expected, the sample shows no clear overall pattern of the effects of changes in uncertainty regarding fertility intentions (Table 2). Women with medium- or low-education levels tend to abandon their fertility plans more often when their male partners experience a rise in employment uncertainty (33.3%), but this does not hold to the same extent for men vis-à-vis their female partners’ rise in employment uncertainty (15.2%). In interpreting these results, we should bear in mind that earlier studies attributing the main breadwinning function to men have clearly associated male employment, not female employment, with fertility intentions and behaviour.

**Table 2:** Description of the sample according to demographic and socio-economic characteristics measured at wave *n*: men (aged 22–50) and women (aged 22–45)

	Intended parents		Stable yes		Abandoners		Postponers		Stable no	
	L–M	H	L–M	H	L–M	H	L–M	H	L–M	H
Employment uncertainty (%)										
Rise	21.6	21.3	16.5	15.6	15.2	21.4	12.8	12.5	13.9	15.8
Decline	14.6	14.0	10.3	8.1	14.7	39.3	17.5	21.9	12.2	19.4
Employment uncertainty partner (%)										
Rise	30.4	31.9	22.1	12.9	33.3	24.1	12.8	19.4	17.7	8.3
Decline	20.4	10.1	13.7	24.2	16.7	17.2	18.9	25.0	18.7	10.5
Year of the first interview										
2002–2003	31.9	35.1	20.9	32.9	34.5	17.6	37.5	32.5	44.1	43.8
2004–2006	40.4	36.8	49.2	39.3	41.4	35.3	37.5	42.5	39.1	39.0
2007–2009	27.7	28.1	29.9	27.8	24.1	47.1	25.0	25.0	16.8	17.2
Age groups (%)										
22–30 years old	42.1	22.3	44.3	35.8	32.4	13.8	47.5	43.8	12.9	7.7
31–40 years old	53.5	73.4	49.4	58.2	58.8	79.3	42.5	50.0	47.1	41.1
41–50 years old	4.4	4.3	6.3	6.0	8.8	6.9	10.0	6.3	40.0	51.1
Sex (%)										
Men	46.5	53.2	48.1	53.7	32.4	79.3	57.5	53.1	46.0	74.5
Women	53.5	46.8	51.9	46.3	67.6	20.7	42.5	46.9	54.0	25.5
Parity (%)										
Zero	50.9	46.8	60.8	73.1	29.4	37.9	67.5	68.8	25.2	25.7
One	31.6	44.7	30.4	20.9	44.1	41.4	17.5	6.3	15.2	13.2
Two to eight	17.5	8.5	8.8	6.0	26.5	20.7	15.0	24.9	59.6	61.1
Income CHF (rounded mean)										
Individual CHF	50,250	76,796	55,245	70,719	49,503	92,093	50,962	63,423	52,830	86,851
Household CHF	985,27	134,339	105,472	126,716	981,25	133,139	995,13	126,501	105,841	134,320

L–M, low–medium education; H, high education; CHF, Swiss Francs

#### Education

Another important variable for our analyses is the respondents’ level of education. This variable is based on the highest level of education achieved, and distinguishes a low level of education (incomplete compulsory school, compulsory school, elementary vocational training, domestic science course, 1-year school of commerce, or a general training school), and a medium level of education (apprenticeship, technical or vocational school, full-time vocational school, bachelor/maturity, vocational high school with a master certificate, or a federal certificate), from a high level of education (vocational high school, university, or academic high school).^4^ Table 2 presents sample statistics separately for the group with a medium or low level of education and the group with a high level of education. Sample statistics indicate that high-education levels seem to matter in the relationship between employment uncertainty and fertility intentions: 39.3% of highly educated men whose partners’ employment situations improve abandon their intentions to have a child, while only 14.7% of men with medium or low levels of education revise their plans.

### Control Variables

We controlled for confounding factors by including age (continuous variable), and yearly household income net the deductions of social security contributions but without tax deductions (Kuhn 2009). We also controlled for parity categorized as 0, 1, or 2 or more, following Yamaguchi and Ferguson (1995), because first-time and second-time childbearing intentions tend to be realized more often compared with third-time and subsequent childbearing intentions (see also Berrington 2004). Finally, we included controls for period effects [i.e. years 2002–2003 are the reference group, period 2004–2006, and (economic crisis) period 2007–2009]. Table 2 shows sample statistics by education and intention–realization type. We observe that the *stable yes* group is the youngest and the *stable no* group the oldest (e.g. 6% are over age 40 in the *stable yes* group versus more than 40% in the *stable no* group). The widest income range is found among those who abandon their fertility intentions (the *abandoners)*, and high incomes correlate with high-education status. Individuals who already had two children had fewer subsequent childbearing intentions (e.g. less than 9% of the *stable yes* group). Finally, one in three low- to medium-educated respondents and one in four highly educated respondents abandoned the intention to have a child if there was a rise in their partner’s employment uncertainty. Interestingly, we also observe that highly educated respondents whose partners’ employment uncertainty declines intend to have a child in wave *n* + 2.

### Analytic Strategy

We used multinomial logistic regression analysis to associate changes in employment uncertainty with the probability that individuals develop one of the intention/(behaviour) trajectories described in Sect. 4.2. This trajectory approach is consistent with our conceptual framework of short-term fertility intentions, defined as concrete childbearing plans for the 24 months following respondents’ reports on their intention to have or not to have a child. This approach estimates the probability that a person *abandons*, *postpones*, or *maintains* the intention to have a child, does *not intend* to have a child at all, or *realizes* an existing intention in the given time frame. Similar approaches have been used in previous research on short-term fertility intentions and subsequent behaviour (Berrington 2004; Spéder and Kapitány 2009; Heaton et al. 1999). Because individuals experience their lives not only as a sequence of events and changes (i.e. of intentions), we also employ a holistic approach to estimate the probability of intentions not changing in the short term. In a low-fertility context like Switzerland, those who consistently do not intend to have a child represent a large portion of the population in reproductive ages; those who do not progress from the intention to have a child to its realization are of interest, as they may actually delay childbearing. In sum, a multinomial approach is the best way to address the patterns of intention/behaviour under focus in this paper, also considering how well it handles the issue of panel attrition. More sophisticated computational techniques, including event history models, should be used when the focus is specifically on intention change and birth outcomes (Allison 2009).

We run separate models for populations with medium and low levels of education as well as for those with a high level of education. We estimated a Chow test for logistic regression (De Marris 2004) to verify whether estimating separate models for each educational subgroup (Tables 3 and 4) would be more informative than the estimations included in the model based on the overall population, where education was only a dummy variable (Table 5 in Appendix). The Chow test, which compares the likelihood estimated for the overall model with the sum of likelihoods estimated for each separate model, indicated that separate estimations fit the data the best.

**Table 3:** Model 1: Multinomial regression predicting effects of employment uncertainty, controlling for socio-demographic variables, on fertility intentions and fertility intention–realization among the *high-education* group; beta coefficients

	Intended parents		Stable yes		Abandoners		Postponers	
	*B*	Sig.	*B*	Sig.	*B*	Sig.	*B*	Sig.
Explanatory variables								
Male employment uncertainty (ref. stability)								
Decline	−0.199	0.684	−0.737	0.158	0.231	0.688	−0.426	0.521
Rise	0.575	0.205	−0.526	0.343	1.278	0.010	0.584	0.313
Female employment uncertainty (ref. stability)								
Decline	1.062	0.023	0.726	0.181	1.707	0.004	1.286	0.029
Rise	−0.043	0.938	1.161	0.017	1.374	0.028	1.068	0.056
Control variables								
Age	−0.234	0.000	−0.170	0.000	−0.141	0.001	−0.244	0.000
Parity (ref. 2 or more children)								
0 child	1.044	0.054	2.099	0.001	1.054	0.091	0.722	0.199
1 child	3.127	0.000	2.640	0.000	2.324	0.000	0.189	0.820
Income CHF (log)								
Individual	0.553	0.152	0.168	0.670	−0.080	0.870	0.206	0.661
Household	0.592	0.293	0.252	0.676	0.774	0.288	0.442	0.550
Sex (ref. women)	−0.449	0.362	0.277	0.563	1.101	0.155	−0.111	0.855
Year of the first interview (ref. 2002–2003)								
2004–2006	−0.097	0.797	0.726	0.104	0.278	0.570	−0.437	0.368
2007–2009	0.229	0.611	1.134	0.020	0.263	0.657	−0.275	0.636
Constant	−7.682	0.153	−3.066	0.592	−8.298	0.234	−1.732	0.802

The reference group is the *stable no* group. *R* ^2^ = 0.465 (Nagelkerke). Model *X* ^2^ (48) = 286.389, *p* ≤ 0.001

**Table 4:** Model 2: Multinomial regression predicting the effects of employment uncertainty, controlling for socio-demographic variables, on fertility intentions and fertility intention–realization among the *low- and medium-education* groups; beta coefficients

	Intended parents		Stable yes		Abandoners		Postponers	
	*B*	Sig.	*B*	Sig.	*B*	Sig.	*B*	Sig.
Explanatory variables								
Male employment uncertainty (ref. stability)								
Decline	−0.763	0.136	−0.112	0.797	0.301	0.644	−0.601	0.284
Rise	−0.540	0.261	−0.586	0.219	0.201	0.757	−0.530	0.335
Female employment uncertainty (ref. stability)								
Decline	0.263	0.522	0.348	0.395	0.752	0.155	−0.295	0.657
Rise	0.136	0.713	−0.690	0.133	−0.185	0.753	−0.178	0.737
Control variables								
Age	−0.155	0.000	−0.141	0.000	−0.103	0.013	−0.142	0.000
Parity (ref. 2 or more children)								
0 child	1.628	0.000	2.446	0.000	0.230	0.711	1.557	0.003
1 child	2.344	0.000	3.028	0.000	1.536	0.005	1.312	0.038
Income CHF (log)								
Individual	−0.053	0.857	0.206	0.550	0.943	0.059	0.032	0.938
Household	0.105	0.824	0.287	0.572	−1.380	0.037	−0.061	0.916
Sex (ref. women)	0.651	0.124	0.386	0.365	−0.854	0.193	1.060	0.041
Year of the first interview (ref. 2002–2003)								
2004–2006	−0.244	0.4830	−0.534	0.137	0.502	0.391	0.055	0.902
2007–2009	0.331	0.389	0.286	0.452	1.559	0.007	0.467	0.349
Constant	1.116	0.804	−4.652	0.345	5.356	0.397	1.060	0.041

The reference group is the *stable no* group. *R* ^2^ = 0.351 (Nagelkerke). Model *X* ^2^ (48) = 256.285, *p* ≤ 0.001

**Table 5:** Model 3: Multinomial regression predicting the effects of employment uncertainty, controlling for socio-demographic variables including education, on fertility intentions and fertility intention–realization; beta coefficients

	Intended parents		Stable yes		Abandoners		Postponers	
	*B*	Sig.	*B*	Sig.	*B*	Sig.	*B*	Sig.
Explanatory variables								
Male employment uncertainty (ref. stability)								
Decline	−0.511	0.133	−0.328	0.320	0.197	0.638	−0.526	0.212
Rise	−0.055	0.861	−0.521	0.146	0.846	0.022	−0.053	0.891
Female employment uncertainty (ref. stability)								
Decline	0.568	0.057	0.412	0.199	1.128	0.003	0.368	0.377
Rise	0.098	0.746	0.079	0.804	0.458	0.265	0.351	0.348
Control variables								
Level of education (ref. high)								
Low–medium	−0.564	0.015	−0.364	0.123	−0.665	0.040	−0.170	0.146
Age	−0.180	0.000	−0.143	0.000	−0.118	0.000	−0.142	0.000
Parity (ref. 2 or more children)								
0 child	1.342	0.000	2.285	0.000	0.574	0.180	1.174	0.002
1 child	2.682	0.000	2.776	0.000	1.817	0.000	0.741	0.125
Income CHF (log)								
Individual	0.201	0.381	0.145	0.557	0.480	0.169	0.103	0.730
Household	0.313	0.387	0.299	0.422	−0.525	0.288	0.130	0.770
Sex (ref. women)	0.236	0.449	0.285	0.362	−0.464	0.984	0.601	0.119
Year of the first interview (ref. 2002–2003)								
2004–2006	−0.166	0.509	−0.017	0.950	0.323	0.380	−0.135	0.874
2007–2009	0.283	0.325	0.578	0.047	1.026	0.010	0.140	1.151
Constant	−2.532	0.471	−3.836	0.284	1.135	0.812	−0.033	0.994

The reference group is the *stable no* group. *R* ^2^ = 0.368 (Nagelkerke). Model *X* ^2^ (52) = 486.879, *p* ≤ 0.001

## Results

### Changes in Employment Uncertainty

Our multinomial logistic regression focused on how changes in both partners’ employment uncertainty are associated with revisions of fertility intentions or their realization. Results of multinomial logistic regression analyses among the highly educated population are shown in Table 3 and among the medium- to low-educated respondents are presented in Table 4.

The association between abandoning an intention to have a child within two years and a rise in employment uncertainty is positive and statistically significant for respondents with a high level of education (see Table 3). Respondents who experience a rise in uncertainty are more likely to abandon an intention to have a child than respondents who do not experience such a rise. In the case of respondents with medium or low levels of education, the association is not significant. The association is positive and significant for both male and female employments. Moreover, the association between intending to have a child over the subsequent 24 months when at the same time employment uncertainty rises is positive and significant as far as women are concerned (*B* = 1.161; *p* ≤ 0.05). The conclusion is therefore that Hypothesis (1a) holds true for highly educated respondents, which thereby also confirms Hypothesis (3a). Hypothesis (4a) is not supported: we expected significantly stronger associations for male than female uncertainty on postponement and abandonment, but did not find any evidence of this. The confidence interval of the odds ratio of a rise in male uncertainty falls within the one of a rise in female uncertainty. In summary, a rise in highly educated men’s and women’s employment uncertainty facilitates an abandonment of the intention to have a child within two years.

The association between the construction of an intention to have a child within two years, that is, a change from not intending to definitely intending to have child, and a *decline* in employment uncertainty is positive and statistically significant for highly educated women. Hence, an improvement of the female partner’s employment situation is conducive to the construction of childbearing intentions. Hypothesis (1b) holds true, therefore, for female employment, but not for male employment. We found similar effects in our base model (see Table 5, Model 3 in the Appendix). Paradoxically, a decline in uncertainty can also have the opposite effect of making respondents more likely to abandon childbearing intentions. This finding suggests that an improvement in the employment situation of women has a more complex influence on childbearing intentions than providing options and resources to achieve fertility goals.

The last possible outcome of our dependent variable is the intention/behaviour trajectory ‘intending a child and childbearing during the next two years’. The model coefficients reflect associations described by Line 2 in Fig. 1 and are reflected in Hypotheses (2a), (2b), and (5a). In the case of a rise in employment uncertainty, we find no statistically significant coefficients; thus, Hypotheses (2a) and (5a) remain unconfirmed. We find, however, a decline in female employment uncertainty to be positively associated with the realization of a childbearing intention. Hence, for female employment uncertainty, Hypothesis (2b) holds, and experiencing an improvement in her employment conditions is indeed conducive to forming childbearing intentions.

Table 4 shows the results for the respondents with a medium level or low level of education. No statistically significant effects of changes in employment uncertainty are found, whatsoever. This lack of significance is interesting but supports Hypothesis (3b) that populations with a medium or low level of education are less responsive to employment uncertainty. Instead, household income is a significant constraint associated with the abandonment of fertility intentions among these respondents. Contrary to the results obtained for the highly educated group, our findings suggest that a medium level or low level of education makes people generally more vulnerable to facing material constraints if they have children, but that the mere experience of employment uncertainty is less harmful in this respect.^5^

**Fig. 2:**
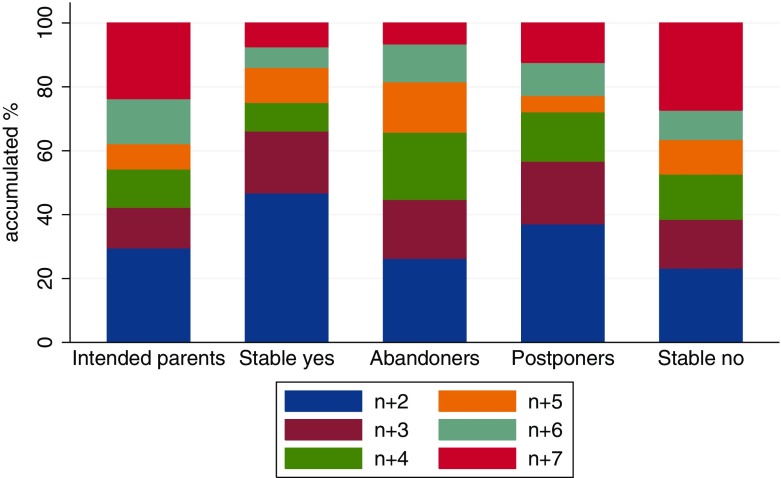
Labour force participation in subsequent years (within group percentages)

**Fig. 3:**
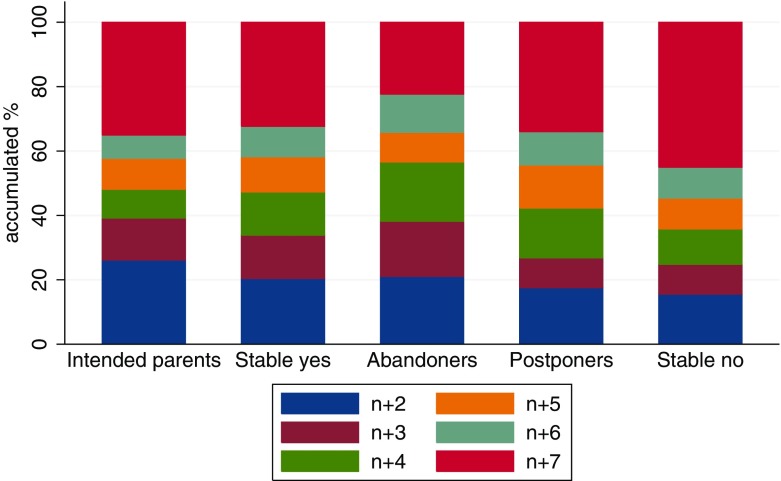
Intention to have no child in subsequent waves (within group percentages)

In a last step, we examined whether there were differences in labour force participation among the five intention revision/realization types. We compared those who abandon their intentions with those who maintain their intentions over the five years following our observation period. Our results indicated dissimilar patterns of labour market attachment between these two groups (Table 6 and Fig. 2 in the Appendix): those who maintain their intentions to have children appear to temporarily interrupt their careers (most probably due to childbearing) and return to employment directly afterwards. Within this group, 20% are employed at the end of the 24-month period (i.e. time 0 on the horizontal axis), while this percentage reduces in each of the two subsequent years, reaching its lowest level in year four (9.5%), and recovering to 32% in year five. Conversely, more of those who abandon their intentions are employed in the first 3 years. Moreover, fewer of those abandoners return to work and do so at a slower pace compared with those who maintain their childbearing intentions (22% among *abandoners* vs. 32% among the *stable yes* group in year five). To verify this, we also looked at intention trajectories for each intention–realization type over five consecutive years (Table 7 and Fig. 3 in Appendix). Fewer *abandoners* intend to have a child in five consecutive years compared with the *stable yes* group. Variation in intentions in the *stable yes* group may indicate that people in this group realized their intentions, which also explains the similarity of their pattern to that of the *intended parents* group compared with those who abandon their intentions.

**Table 6:** Number of times respondents participate in the labour force in each group

In % (columns)	Intended parents	Stable yes	Abandoners	Postponers	Stable no
0	26.12	20.40	21.05	17.53	15.53
1	13.06	13.43	17.11	9.28	9.26
2	8.93	13.43	18.42	15.46	11.00
3	9.62	10.95	9.21	13.40	9.58
4	7.22	9.45	11.84	10.31	9.58
5	35.05	32.34	22.37	34.02	45.05

**Table 7:** Number of times respondents do not intend to have a child in each group

In % (columns)	Intended parents	Stable yes	Abandoners	Postponers	Stable no
0	29.55	46.77	26.32	37.11	23.24
1	12.71	19.40	18.42	19.59	15.28
2	12.03	8.96	21.05	15.46	14.17
3	7.90	10.95	15.79	5.15	10.81
4	14.09	6.47	11.84	10.31	9.19
5	23.71	7.46	6.58	12.37	27.31

### Patterns Across Groups

We now look at patterns in fertility intentions and fertility behaviour across populations with a low or medium level of education and those with a high level of education, and find major common patterns in the socio-demographic factors (see Tables 3, 4). Most importantly, we find statistically significant period effects for 2007–2009 when the economic downturn fuelled unemployment rates to reach unprecedented levels in Europe, and created a substantive feeling of uncertainty among the population in Switzerland. While an increase in individual employment uncertainty makes highly educated respondents more likely to abandon an intention to have a child within two years (we recall that Hypotheses (1a) and (3a) are confirmed), general uncertainty during economic downturns increases the likelihood that this group delays realization by potentially waiting until better times arrive. In the case of respondents with medium or low levels of education, the general uncertainty rather than the personal employment situation is what increases their probability to abandon an intention to have a child. Effects of other characteristics do not change when introducing period controls (results will be provided upon request).

The multinomial models show consistently that, with age, the construction of an intention to have a child and the realization of such intention decline. Apart from age, parity is a main criterion for fertility intentions and fertility behaviour. Having no or one child is strongly associated with the intention to have (an additional) one in both populations. However, if we compare those people who postpone their intention to those who consistently reject the idea of childbearing, it appears that only people with a low or medium level of education with no or one child are significantly more likely to postpone having a(nother) child relative to rejecting this intention. Parents of one child are more likely in both populations to realize their intention to have a second than to consistently reject the idea of having a child.

## Discussion and Conclusion

This study explored changes in employment uncertainty, the formation of fertility intentions, and the realization of such intentions among working couples living in Switzerland. We have tested two situations: a rise in employment uncertainty and a decline in employment uncertainty. We have formulated three arguments, concerning (1) the association between employment uncertainty effects and fertility intentions, (2) their realization, and (3) differences in these associations by education. We checked the hypotheses in the context of Switzerland, where a highly liberal labour market and poor work–family reconciliation policies (Armingeon 2001; Armingeon et al. 2004) and gendered parenting norms (Bühlmann et al. 2016) translate into high levels of childlessness and low fertility (Sobotka 2011). A focus on the Swiss context is relevant, because employment uncertainty may reflect worries of losing a job even if on a permanent contract, or it may be due to worries of not being able to reconcile working with childrearing and care duties. We found that the results are specific according to education levels. The summary is presented first for the intention trajectories and subsequently for the intentions and actual childbearing.

We confirm our *intention*-*trajectory argument (1)*: Worsening employment conditions of men and women facilitate abandonment, and women’s worsening employment conditions motivate postponement as well. However, the association between women’s improved employment conditions and their fertility intentions is less straightforward, because these conditions facilitate either the construction or the abandonment of an intention to have a child. An improvement of women’s employment situation can either indicate an increase in options and resources for them to achieve fertility goals (for instance privileges tied to permanent contracts), or it can indicate strong involvement in the labor market, which competes with childrearing and care duties. These associations hold true only for respondents with a high level of education. Indeed, the relationship between childbearing and employment may be moderated by education. More than 20% of highly educated women in Switzerland remain childless (Sobotka and Zeman 2011); there is a strong trend to choose higher involvement in lifelong employment over a commitment to raising children. These women often live in educationally homogamous couples and experience lengthy employment episodes due to pooling their resources compared to their less educated peers (Blossfeld and Timm 2003).

This finding is an extension of the pioneering work of Spéder and Kapitány (2009), which was based upon the effects of unemployment on fertility intentions trajectories. The results reported here support their assumption that using a more refined indicator than a simplified activity status might help to better understand effects of women’s employment conditions on fertility decision-making. In our case, the observed trajectory of postponement can be viewed as resulting from a tendency to commit fully to work in order to establish a strong labour market position—a priority especially among highly educated women—which often competes with their commitment to other life domains, such as responsibly caring for a child. The reported results also indicate a modernized ‘breadwinner model’ where men and women both engage in paid labour and, therefore, their time and energy are compromised by the task of caring for their child(ren) (Hochschild and Manchung 1989).

We confirm the *intention*–*realization argument (2)*: women’s improved employment conditions are conducive to childbearing. Men’s improved employment conditions are not found to encourage childbearing, even if an intention to have a child were to exist. No statistically significant associations were found for the realization of an intention to have a child and worsening employment conditions.

These findings suggest that childbearing depends upon women’s employment conditions, the potential opportunity costs, and possibilities of balancing time and energy spent at work and in the home. The results hold only true for respondents with a high level of education.

We confirm the *education argument (3):* changes in employment conditions [*(1)* and *(2)*] have a strong impact on the relationship between fertility intention and realization among highly-educated individuals, but no significant impact among low- and medium-educated individuals.

Employment uncertainty hampers childbearing intentions among the highly educated population, which suggests that opportunity costs of childbearing and the efforts related to responsibly caring for a child play a major role. On the contrary, among medium- and low-educated populations, material constraints hamper childbearing intentions from being realized. The latter result lends support to Kohler and Kohler’s argument (2002) that men’s unstable employment, thus uncertain income, hampers fertility. Likewise, a higher unemployment rate during an economic crisis hampers the realization of intentions among the highly educated population and makes individuals with medium or low levels of education more likely to abandon the idea of childbearing. A potential explanation is that the former delay childbearing until employment conditions improve, whereas the latter give up on the idea altogether due to the material deprivation they face.

Some limitations of this study should be mentioned. First, this study is limited to short-term fertility intentions. Since they neither inform about the intended number of children nor capture the entire reproductive lifespan of the respondent, it remains unclear whether postponers or those who maintain their intention to have a child actually achieve their goal; we are also aware that our measurement of intentions over a 24-month period may not be sufficient to grasp variations in intentions in the very short run; neither did it allow insight into sudden intention change due to critical events (e.g. an acute illness). The analytical choice of our time frame, however, allows us to capture the capacity to form concrete childbearing plans in the near future. Second, dynamic approaches like event history techniques should be used to predict the timing of intention change and births. Given that lives are not solely experienced as a sequence of changes and events, we chose a holistic approach to estimate fertility intention/(behaviour) trajectories as well as to be able to assess the probability that individuals do not change an intention to have or not to have a child. Such an approach is especially valuable in low-fertility contexts where large parts of the reproductive-age population postpone childbearing, and therefore, individuals who continue to intend to have a child are most likely delaying having children. This approach was also the best way to handle our data, specifically the panel attrition within our sample of reproductive-age individuals. Third, this study examines the experienced employment uncertainty and the one related to limited-term contracts, as these are the best available indicators for employment uncertainty in our dataset. The inclusion of transitions into and out of unemployment would have been interesting, although differences in fertility intentions of the employed and unemployed are more likely driven by the conjugal situation, the level of education, and migratory background (Pailhé and Solaz 2012). The number of observations—both unemployed respondents and their transitions into or out of unemployment—is too low to warrant their inclusion into the studied sample. Moreover, the reciprocal influence of partners on each other’s fertility intentions (Testa et al. 2014; Cavalli and Rosina 2011) and the higher risk of disagreement among highly educated couples (Rosina and Testa 2009) would have motivated a couple intention analysis, but our focus on intention trajectories and intentions and their realization did not warrant such a strategy due to small numbers. We note that in the current analysis the numbers of respondents in the highly educated group are small in some categories, such as the abandoners and the postponers group. Finally, future work should address employment uncertainty throughout one’s lifespan and in relation to other life domains and stages in order to account for people’s actual priorities as well as priority shifts that matter for reproductive decisions.

Despite such limitations, our results have a number of implications for our understanding of the nuances of the link between employment uncertainty and fertility intentions and their realization. First, although few differences in the effect on fertility with respect to a rise in uncertainty were observed by gender, major gender differences in the association of a male versus a female uncertainty decline were found. This suggests that in contrast to men, women—especially highly educated women—still seem to be confronted with the choice between having a career and investing in the labour market (to reduce their employment uncertainty) or having a child. Second, fertility intentions/behaviour trajectories varied considerably between educational groups, and a few socio-economic differences in the uncertainty–fertility link were observed. This suggests that one should not juxtapose multiple socio-economic groups but should rather account for the variety of educational backgrounds and how these influence their reproductive decisions. Third, our analysis showed that childbearing intentions and their realization are related to subjective evaluations on the future labour market participation; those who abandon the intention to have a child do so because their labour market prospects are bleak. Overall, we show that if reproductive decisions vary by educational group, they also vary within educational groups, according to individuals’ labour market perspectives and their partners. Our findings are conservative, since we are analysing the Swiss context in which, despite increased feelings of economic insecurity, unemployment, and labour market uncertainty are low. In contexts of higher unemployment and labour market uncertainty, social inequalities by gender, education, and access to the labour market may play a larger role in determining who is able to realize childbearing intentions and who is not.

## Appendix

See Tables 5, 6 and 7 and Figs. 2 and 3.

## Footnotes

1 Respondents who may have changed their fertility intentions cannot be properly distinguished from those having unintended births. Such an undetectable change is a common limitation in longitudinal studies with a 24-month gap between one measurement and the other.

2 Unlike in countries such as Great Britain or the USA, which score higher on unintended births, Switzerland has a high level of contraceptive use and family planning centres are especially sensitive to the younger population, effectively keeping the teenage pregnancy rate low. For many Swiss women and couples, the main issue is not to avoid unintended births, but rather when to stop contraception and how to time an intended pregnancy.

3 Assessing employment uncertainty among permanent employees is important, especially where liberal national employment protection legislation does not strictly protect employees under permanent contracts from being dismissed or laid off (OECD 2015).

4 Different educational attainment types are matched under the medium–low and high-education rubrics, so that one may argue that the education variable is an oversimplification of the diversity of existing educational credentials and a more differentiated analysis should be performed (Adsera 2011). In our data, too few people had very low education to be analysed separately. Furthermore, exposing the effects for each educational credential separately would have been beyond the scope of this paper.

5 Analyses (not shown) by parity yield less pronounced results, but suggest that a rise in male employment uncertainty is associated with abandonment and that parenthood reduces the fertility effects of female employment.
